# Notched noise reveals differential improvement in the neural representation of the sound envelope

**DOI:** 10.1038/s42003-025-08536-4

**Published:** 2025-08-07

**Authors:** Amarins N. Heeringa, Jonas Klug, Christine Köppl, Mathias Dietz

**Affiliations:** 1 Department of Neuroscience, School of Medicine and Health Science, Carl von Ossietzky University Oldenburg, Oldenburg, Germany; 2 Research Center Neurosensory Science, Carl von Ossietzky University Oldenburg, Oldenburg, Germany; 3 Cluster of Excellence “Hearing4all”, University of Oldenburg, Oldenburg, Germany; 4 Department of Medical Physics and Acoustics, Carl von Ossietzky University Oldenburg, Oldenburg, Germany

**Keywords:** Transduction, Neurophysiology, Neural encoding

## Abstract

Precise temporal coding in auditory nerve fibers is crucial for sound localization and listening in noise. However, at higher sound levels, typical of everyday listening situations, temporal coding to the stimulus envelope is poor in fibers of the on-frequency channel, i.e., those tuned to the carrier stimulus. We predict that changes in cochlear gain improve temporal coding of the stimulus envelope differentially across frequency channels. Both auditory nerve fiber recordings (in Mongolian gerbils of either sex) and human psychophysics confirm that weak temporal coding at higher levels is improved when the target stimulus is presented in a spectrally flanking notched noise designed to reduce the cochlear gain. The proposed mechanism can help to explain the effect of cochlear gain loss, a known consequence of age- and noise-induced hearing loss, on everyday listening, such as problems with speech-in-noise perception and sound localization.

## Abstract (web summary)

Auditory nerve recordings, human psychophysics, and a computational model consistently indicate that stimulus-induced changes in cochlear gain improve temporal coding of the stimulus envelope at intermediate and high sound levels.

## Introduction

Hearing in noise requires many processing steps at all levels of the auditory system^1^. Sensory encoding by the hair cells of the cochlea and the subsequent encoding by the auditory nerve fibers lays the crucial foundation. For instance, speech-in-noise performance correlates strongly with the function of the outer hair cells^2^. In a healthy cochlea, outer hair cells are the driving force in a positive feedback loop that amplifies sound-induced basilar membrane vibrations^3^. This cochlear amplification acts in a frequency- and level specific way and is under efferent control^4^. It improves the operating point and spectral resolution for the afferent pathway, starting with the inner hair cells and the auditory nerve fibers connecting to the central auditory system^5^. At low sound levels, outer hair cell electromotility improves the sensitivity of auditory nerve fibers, whereas at high sound levels the cochlear gain relative to the movement of the middle ear is a few orders of magnitude lower^6^. Dallos and Harris^5^ were the first to suggest that cochlear gain may influence phase locking of auditory nerve fibers as well. Phase locking, usually quantified as vector strength (VS), describes the ability of neurons to precisely time their spiking to temporal fluctuations of a sound waveform. Phase locking to the slowly fluctuating stimulus envelope is of crucial importance for speech-in-noise encoding^7–9^ and can serve as the sole cue for speech recognition^10^. Hence, by modulating phase locking of auditory nerve fibers to the speech envelope, outer hair cells could support speech-in-noise processing.

The sinusoidally amplitude-modulated (SAM) tone, comprised of a simple tone (the carrier) that is modulated by a much lower-frequency sinusoid, is a standard stimulus for testing neural envelope phase locking. In auditory nerve fibers, phase locking to the envelope of an SAM tone first increases and then decreases with increasing sound pressure level (SPL)^11–15^, due to saturation of the receptor potential of the inner hair cell and of the firing rate throughout most of the envelope modulation cycle^16,17^. Here, we hypothesize that noise in the spectral proximity of an amplitude-modulated sound, which causes suppression and thus outer hair cells to reduce the effective cochlear gain^18^, restores phase-locking to the envelope at moderate sound levels in fibers tuned to the carrier frequency, the so-called on-frequency channel. This hypothesis is already implicitly included in some of the comprehensive computational models of the inner ear^19,20^. Its functional consequences have only recently been conceptualized and simulated with an amplitude-modulated tone in notched noise (noise that is specifically missing the frequency range around the carrier frequency)^21,22^. With the use of the above-mentioned models, both studies predicted that notched noise reduces firing rate and increases phase locking in the on-frequency channel.

Here, we investigate the dynamics of this phenomenon by using a computational model of the inner ear and afferent pathway, and test our predictions experimentally by recording in vivo from single-unit auditory nerve fibers in an animal model, the Mongolian gerbil. To translate the hypothesis to human hearing, we rely on indirect evidence, due to the inaccessibility of auditory nerve fibers in humans. A common proxy of the encoded temporal envelope information in the human auditory system is to psychoacoustically test the sensitivity to interaural time difference (ITD). In the absence of notched noise, envelope ITD (ITD_ENV_) sensitivity of humans improves with increasing level^23,24^ suggesting a better representation of the temporal envelope when the sound becomes more intense. This seems in contrast to the above-mentioned decline of auditory nerve phase locking to the envelope with increasing level^11–15^. The parsimonious explanation for this apparent contradiction may be that the auditory nerve studies focused on fibers tuned to (preferring) the carrier frequency, whereas the subjects in the psychoacoustic studies can access temporal information across the full tonotopic array, including neurons in so-called off-frequency channels^25^. These neurons may provide complementary, superior envelope phase locking at high stimulus levels. This has led to the hypothesis that ITD_ENV_ sensitivity deteriorates when presenting the SAM tones in notched noise, reasoning that this noise masks specifically the responses in off-frequency channels^26^. However, published data do not fully support this hypothesis: ITD_ENV_ thresholds only increased by a factor of 2 in notched noise, suggesting level-robust on-frequency processing even in the presence of notched noise^27^. Our above-mentioned hypothesis, that notched noise improves on-frequency envelope representation at higher stimulus levels, can resolve the apparently conflicting conclusions drawn from physiology vs. psychoacoustics. Specifically, without notched noise, ITD_ENV_ sensitivity is good off-frequency and arguably poor on-frequency, whereas the notched noise shuts down off-frequency sensitivity while improving on-frequency sensitivity.

In the current study, we demonstrate both computationally and experimentally that noise in the spectral proximity of an amplitude-modulated tone improved on-frequency auditory nerve phase locking to the envelope at moderate levels. This was caused by a shift in the operating point, likely mediated by outer hair cells reducing the effective gain. Psychoacoustically, we used the concept that ITD_ENV_ sensitivity requires temporal information to be available within interaurally matched frequency channels^23,28^. When notched noise was presented monaurally, we found that ITD_ENV_ sensitivity deteriorated compared to the binaurally presented notched noise. This suggests a mismatch between frequency channels that carry the temporal information: On-frequency sensitivity at the ear with notched noise but off-frequency sensitivity on the contralateral ear without noise, consistent with our hypothesis.

## Results

### Envelope phase locking was superior in the on-frequency channel at low sound levels, but in the off-frequency channels at high levels

**Fig. 1:**
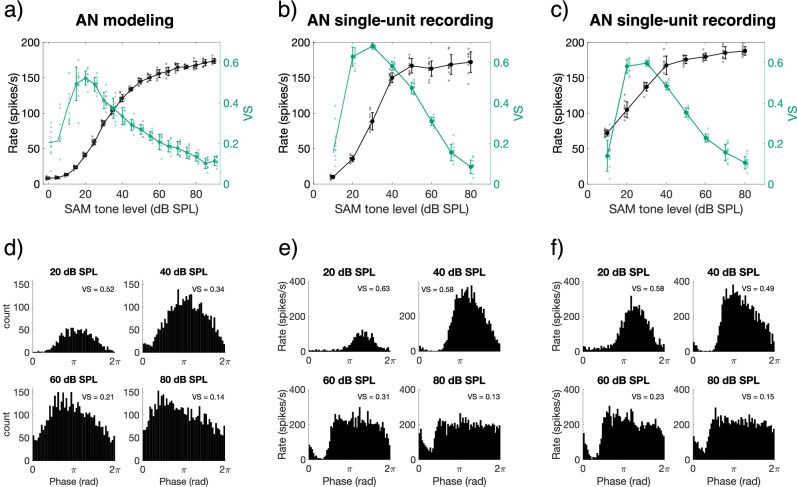
On-frequency phase locking to the stimulus envelope was highest close to threshold. **a** The rate-level and VS-level functions of a modeled fiber in black and green traces, respectively. Only VS values that were statistically significant^62^ were plotted with a solid marker with error bars. The CF of the modeled fiber was 4 kHz and the SAM tone had a f_m_ at 128 Hz and a f_c_ at 4 kHz. **b** Data derived from a gerbil auditory nerve fiber with CF = 4.5 kHz and SR = 11.3 spikes.s^−1^. Data are plotted in a similar way as in panel (**a**). **c** Data derived from a gerbil auditory nerve fiber with CF = 4.1 kHz and SR = 78.5 spikes.s^-1^. Data are plotted in a similar way as in (**a**). Auditory nerve fibers were probed with an SAM tone with f_c_ at CF and f_m_ at 128 Hz. Solid markers with error bars in panels (**a**), (**b**), and (**c**) are means ± SD across 10 repetitions of the SAM tone. Individual data points are shown next to the respective markers. **d**–**f** Phase histograms for f_m_ at 20, 40, 60, and 80 dB SPL SAM tone, corresponding to the modeled and neurophysiological data presented in panels **a** and **b**, respectively. AN auditory nerve, CF characteristic frequency, f_c_ carrier frequency of the SAM tone, f_m_ modulation frequency of the SAM tone, SAM sinusoidally amplitude modulated, SPL sound-pressure level, SR spontaneous rate, VS vector strength.

To explore envelope phase locking to SAM tones at various sound levels and across the tonotopic axis, we used the computational model of the auditory nerve by Bruce, et al.^19^. The carrier frequency (*f*_*c*_) was fixed at 4 kHz and the modulation frequency (*f*_*m*_) at 128 Hz, allowing for a direct comparison to the psychophysical study presented here and to that of Bernstein and Trahiotis^27^. For a modeled fiber tuned at 4 kHz, i.e., the on-frequency channel, phase locking (quantified as VS) was highest at 20 dB SPL, which is 5 dB above the rate threshold (Fig. 1a). Phase locking then slowly decreased with increasing SAM tone level (Fig. 1a), consistent with previous findings in cats^11,12^. A similar relation between VS and level was apparent in two example fibers recorded from a young-adult, normal-hearing Mongolian gerbil, that were tuned at 4.5 kHz and 4.1 kHz, respectively, in response to a 128-Hz SAM tone with *f*_*c*_ at their tuning frequency (Fig. 1b,c). Again, the strongest phase locking occurred at levels close to the fiber’s rate threshold and then drastically decreased with increasing level in both fibers. Phase histograms of both the modeled and the in vivo recorded fibers at 20, 40, 60, and 80 dB SPL illustrated that rate saturation over the modulation period became increasingly apparent at higher stimulus levels. While the phase histogram at 20 dB SPL reveals a clear phase preference of firing, resulting in high VS-values of 0.52–0.63, the spike rates in the phase histogram at 80 dB SPL were more consistently high throughout the modulation cycle (Fig. 1d–f). The modeled phase histograms at the highest level of 80 dB SPL showed a shallow peak, whereas the recorded phase histograms saturated by leaving only a dip in firing rate at a given phase. This resulted in much lower phase locking values in both the modeled and recorded fibers.

To determine whether this level dependence of VS is consistent across different auditory nerve fibers in the gerbil, we recorded on-frequency SAM responses from fourteen gerbil auditory nerve fibers at 40 and 60 dB SPL. For all fibers, the VS was lower at 60 dB SPL compared to 40 dB SPL, with an average decrease in VS of 0.23 ± 0.06 (paired *T*-test: T(13) = 14.03, *p* = 3.14 × 10^−9^), consistent with a decrease in phase locking at higher stimulus levels (Supplementary Fig. 1). Note that the 40-dB SPL SAM tone was above rate threshold for all fibers and that *f*_*c*_ was adjusted to match the fiber’s characteristic frequency (CF), i.e., testing the on-frequency channel.

**Fig. 2:**
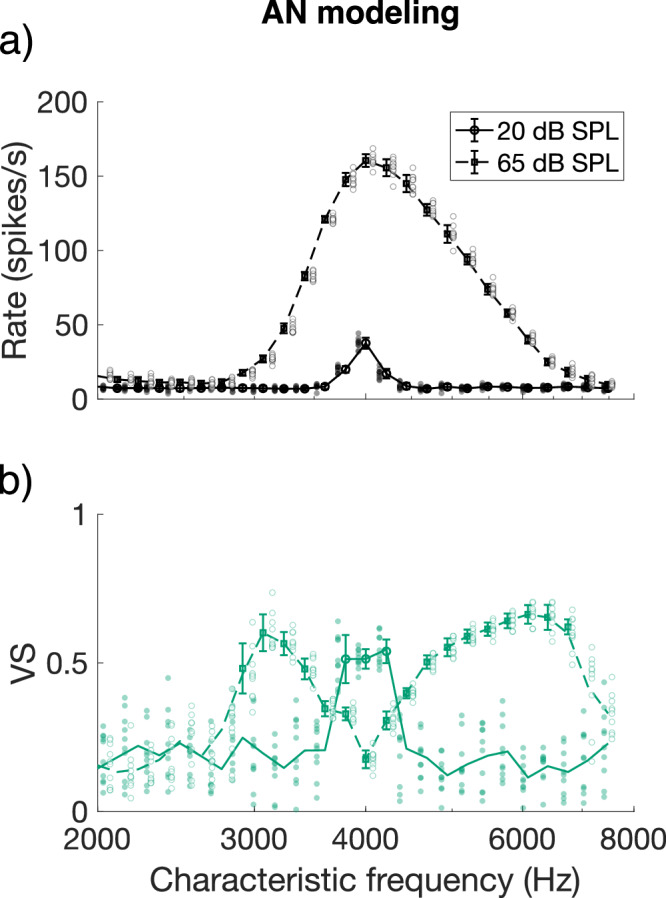
Phase locking to the stimulus envelope was highest in off-frequency channels at higher sound levels. **a** Firing rate as a function of CF for a population of modeled fibers (*n* = 25; one at each CF), in response to a 4-kHz centered SAM tone at 20 dB SPL (squares and solid line) and at 65 dB SPL (circles and dashed line). **b** VS as a function of CF for the same two stimuli as in panel (**a**). The legend of panel (**a**) applies. Only significant VS values were plotted with a marker. The individual data points are shown next to the respective markers and error bars. AN auditory nerve, CF characteristic frequency, SAM sinusoidally amplitude modulated, SPL sound-pressure level, VS vector strength.

To determine phase locking by off-frequency channels, we modeled the responses of auditory nerve fibers with a large range of CFs to an SAM tone with *f*_*c*_ fixed at 4 kHz. At 20 dB SPL (solid line), only fibers in the on-frequency channel increased their firing rate, while at 65 dB SPL (dashed line), a broad range of frequency channels around *f*_*c*_ responded to the SAM tone (Fig. 2a). Correspondingly, phase locking was maximal in the on-frequency channel at the low level, whereas at 65 dB SPL, phase locking was highest about half an octave above and below *f*_*c*_ (Fig. 2b), consistent with previous predictions^21,22^.

### Notched noise improved on-frequency phase locking at high sound levels

**Fig. 3:**
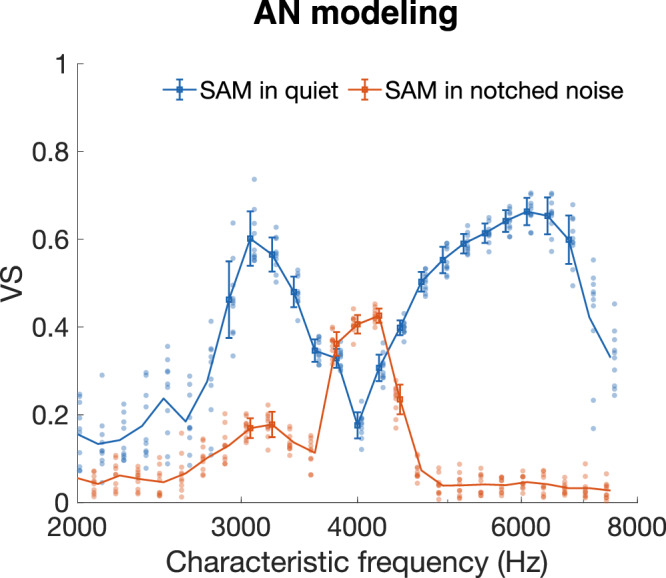
Notched noise improved on-frequency phase locking in modeled auditory nerve fibers. VS as a function of CF for a population of modeled fibers (*n* = 25 fibers; one fiber at each CF) in response to an SAM tone (f_c_ = 4 kHz) in quiet (blue trace) and in notched noise (notched width w = 20% and relative spectral noise level g = −35 dB.Hz^−1^) (red trace). Error bars indicate ± SD. VS values that were statistically significant^62^ are shown with a marker. The individual data points are shown next to the respective markers and error bars. AN auditory nerve, CF characteristic frequency, f_c_ carrier frequency of the SAM tone, SAM sinusoidally amplitude modulated, VS vector strength.

Next, the stimulus of 65 dB SPL was used in the presence of a notched noise with a 20% notch width (*w*) around the *f*_*c*_ of 4 kHz and a spectral noise level of 30 dB SPL.Hz^−1^ (resulting in a relative spectral noise level of *g* = −35 dB.Hz^−1^ relative to the level of the SAM tone). In the model response to this stimulus, the highest VS values were now found in modeled fibers tuned at 4 kHz again, i.e., in the on-frequency channel (see red trace in Fig. 3). Not only was phase locking highest in the on-frequency channel, VS had also increased by 0.23 compared to the VS values in response to the SAM tones without notched noise. These simulations in the model of Bruce, et al.^19^. confirmed our hypothesis, specifically that in the presence of notched-noise, the on-frequency channel improves its ability to represent the temporal envelope.

**Fig. 4:**
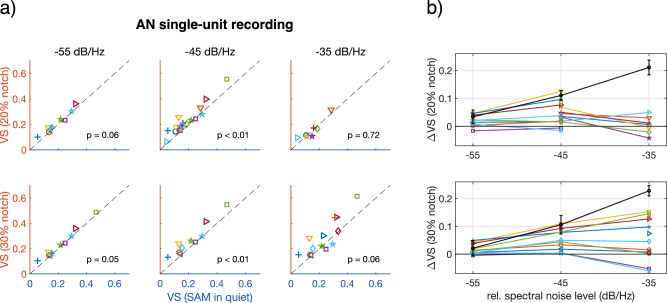
Notched noise improved on-frequency phase locking in auditory nerve single-unit responses. **a** VS to SAM tones with f_c_ = CF, in notched noise, as a function of VS to SAM in quiet (*n* = 17 fibers). Notch width is indicated on the left side of the plots and relative spectral noise level above the plots. Each fiber has a unique marker, that is consistent between all plots. *P*-values in the plots derive from paired *T*-tests and are corrected for multiple comparisons (Bonferroni–Holm). The black dashed line indicates *y* = x, as a visual aid. Data points above this line showed an increase in on-frequency phase locking when notched noise was present. **b** The same data as in panel (**a**), but now represented as ΔVS (VS in notched noise – VS in quiet) as a function of relative (rel.) spectral noise level. Markers correspond to the ones used in panel (**a**). The black trace shows the outcomes from the modeled data for comparison. Data are shown for notch with w = 20% and w = 30% notch widths and are plotted across relative spectral noise level. AN auditory nerve, CF characteristic frequency, f_c_ carrier frequency of the SAM tone, SAM sinusoidally amplitude modulated, VS vector strength.

To test this hypothesis in vivo, we collected responses to SAM tones with and without notched noise from 24 gerbil auditory nerve fibers. Each fiber was characterized according to its CF, response threshold at CF, and spontaneous rate (SR). Furthermore, the auditory nerve fibers were divided between low-SR and high-SR fibers, with a cut-off at 18 spikes.s^−1^^29–31^. Single-fiber responses to 60 dB SPL SAM tones were recorded with and without notched noise. Notched noise with notch widths of *w* = 20% and *w* = 30% around *f*_*c*_, and relative spectral noise levels of *g* = −35 dB.Hz^−1^, −45 dB.Hz^−1^, and, −55 dB.Hz^−1^ were presented, resulting in six different notched noises. The carrier frequency of the SAM tone was closely matched to the individual fiber’s CF, to determine on-frequency envelope phase locking. VS improved for all fibers for at least one of the notched noise conditions (Fig. 4a). Two of the six conditions (notch width *w* = 20% and *w* = 30% with *g* = −45 dB.Hz^−1^) resulted in a statistically significant increase in VS compared to SAM in quiet (paired *T*-tests; T(16) = 4.62, *p* = 1.72*10^−3^ and T(9) = 4.30, *p *= 9.90*10^−3^ for the 20% and 30% conditions, respectively; Bonferroni-Holm corrected; Fig. 4a). The largest increase in VS for an individual fiber (0.15) was observed when SAM tones were presented in notched noise with *w* = 30% (notch width) and *g* = −35 dB.Hz^−1^ (relative spectral noise level), even though the VS increase missed statistical significance (T(12) = 2.604, *p* = 0.062, Bonferroni–Holm corrected; Fig. 4b). In this condition, there were fibers that showed large increases, but also fibers that showed small decreases in VS when the SAM was presented in notched noise. When correlating the notched noise-induced VS change (ΔVS) with the fiber characteristics (CF, threshold, and SR), we found that only SR showed a significant negative correlation with the change in VS (Pearson’s correlation coefficient *ρ* = −0.67, *p* = 0.012; Supplementary Fig. 2). In other words, the effect of *w* = 30%, *g* = −35 dB.Hz^−1^ notched noise on the encoding of the on-frequency stimulus envelope was strongest for fibers with a low SR.

### Notched noise shifted the auditory nerve input-output function

**Fig. 5:**
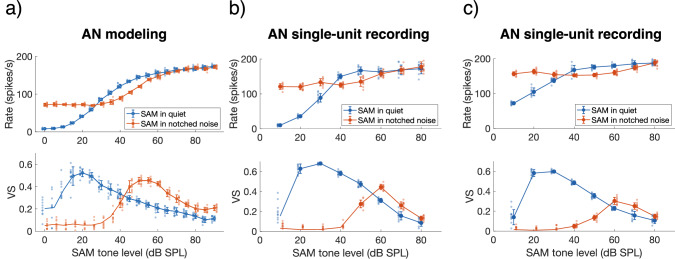
Notched noise shifted the input-output curves. **a** Data derived from a modeled auditory nerve fiber with a CF = 4 kHz and an SR = 4 spikes.s^_^^1^. The rate-level functions (upper panel) and VS-level functions (lower panel) are shown for responses to SAM tones in quiet (blue trace) and SAM tones in fixed-level notched noise (red trace). Solid markers and error bars, represent means ± SD. The individual data points are shown next to the respective markers and error bars. Only statistically significant VS values are shown with a solid marker in the VS-level curve. **b** Data derived from a gerbil auditory nerve fiber with CF = 4.5 kHz and SR = 11.3 spikes.s^−1^. Data are plotted in a similar way as in panel (**a**). **c** Data derived from a gerbil auditory nerve fiber with CF = 4.1 kHz and SR = 78.5 spikes.s^−1^. Data are plotted in a similar way as in panel (**a**). AN auditory nerve, CF characteristic frequency, SAM sinusoidally amplitude modulated, SPL sound-pressure level, SR spontaneous rate, VS vector strength.

The observed improved on-frequency phase locking by notched noise may be associated with a decreased sensitivity of the fiber, possibly due to reduced cochlear gain effected by outer hair cells as evident in suppression^18^. To test this hypothesis, the response rate and phase locking were obtained at a range of different levels of the SAM tone in quiet. This same stimulus was then also presented in notched noise with a fixed level (25 dB SPL.Hz^−1^), resulting in a steadily increasing signal-to-noise ratio with increasing SAM level. This fixed level of 25 dB SPL.Hz^−1^ was selected based on our earlier results, that the −35 dB relative spectral level resulted in the largest changes in ΔVS for SAM tones at 60 dB SPL (see Fig. 4). The response of the modeled fiber showed that the sound level where maximal phase locking was observed increased by about 25 to 30 dB in the presence of notched noise (Fig. 5a). We confirmed this finding in two recorded auditory nerve fibers, both tuned close to 4 kHz, one with a low SR (11 spikes.s^−1^, CF = 4.5 kHz; Fig. 5b) and one with a high SR (78 spikes.s^−1^, CF = 4.1 kHz; Fig. 5c), that were stimulated with on-frequency SAM tones (*f*_*c*_ = CF). Both fibers revealed a rightward shift of the VS-level curve when the SAM tone was presented in notched noise, compared to the same SAM tone in quiet. Note that the effect of notched noise was again stronger on the low-SR fiber (ΔVS_max_ = 0.13) compared to the high-SR fiber (ΔVS_max_ = 0.09).

### Monaural notched noise decreased ITD_ENV_ sensitivity compared to binaural notched noise

After demonstrating an increase in phase locking in single auditory nerve fibers by adding notched noise, we turned to the psychoacoustic consequence explained in the Introduction. We built on previous studies, which showed that ITD_ENV_ sensitivity requires the temporal information from the left and right ear to be available within the same frequency channel^23,28^. If the stimulus envelope is primarily encoded in off-frequency channels in the absence of notched noise and in the on-frequency channel in its presence, as hypothesized, ITD_ENV_ sensitivity should be severely compromised when the notched noise is presented on only one side.

**Fig. 6:**
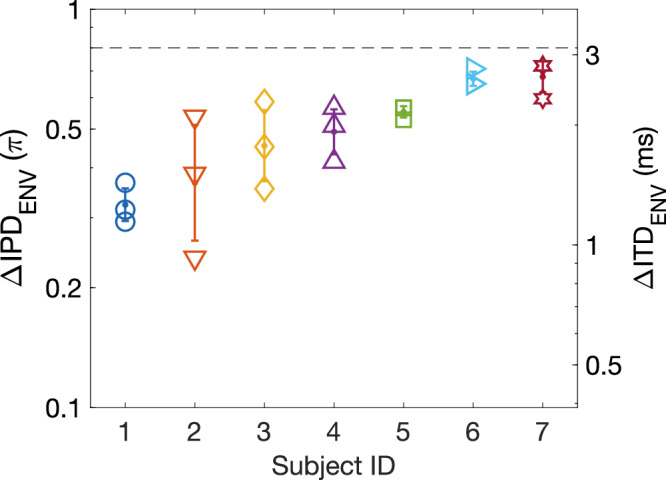
ITD_ENV_ sensitivity to SAM tones presented in binaural notched noise. The ITD_ENV_ sensitivity at a 0.84 proportion correct for each of the seven included subjects. Values are shown as ΔIPD_ENV_ (left axis) and as ΔITD_ENV_ (right axis). The experimental protocol was repeated three times, and the symbols indicate the converted ITD_ENV_ sensitivity from each repetition. The dot and error bars represent the geometric mean and geometric standard deviation, respectively, for each subject. The black dashed line indicates the exclusion criteria, a maximum allowed ΔIPD_ENV_ sensitivity of 0.8π. ITD_ENV_ envelope interaural time difference, ΔITD_ENV_ difference in ITD_ENV_ between two binaural stimuli, ΔIPD_ENV_ difference in envelope interaural phase difference between two binaural stimuli, SAM sinusoidally amplitude modulated.

A prerequisite to demonstrating this deterioration is, of course, a remaining ITD_ENV_ sensitivity when notched noise is presented to both ears. This was measured by a two-alternative forced-choice task, with a ‘four-down, one-up’ staircase procedure. The task of the subject was to indicate whether the SAM tone in the second interval was perceived to the left or the right of that presented in the first interval. The staircase procedure adapted towards a 0.84 proportion correct^32^, to provide a sufficiently large result space for deterioration upon monaurally presented notched noise before a floor is hit at the chance level of 0.5 proportion correct. Furthermore, the resulting ∆ITD_ENV_ threshold needed to be smaller than 3.125 ms (equal to 0.8π interaural phase difference, ∆IPD_ENV_), as a decline of sensitivity is expected again at higher values of ∆ITD^33^. For the seven subjects included in this study, their ∆ITD_ENV_ threshold for SAM tones in binaural notched noise at 0.84 proportion correct differed (Fig. 6) but were all smaller than 0.8π ∆IPD_ENV_.

**Fig. 7:**
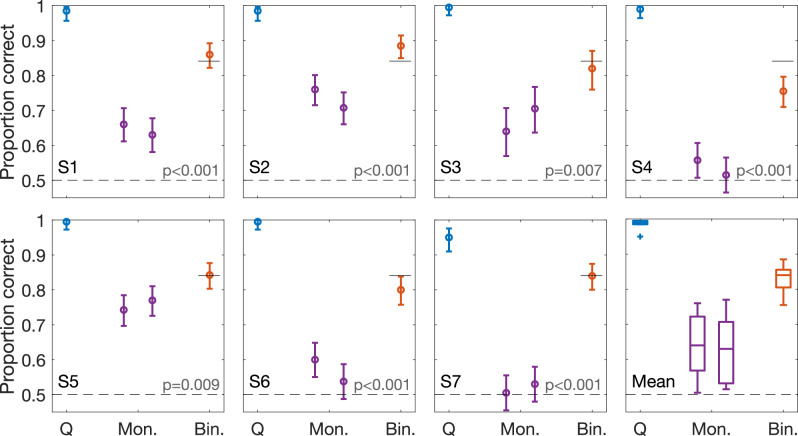
ITD_ENV_ sensitivity in quiet, in monaurally presented, and in binaurally presented notched noise. Proportion correctly localized stimuli when SAM was presented with a ΔIPD_ENV_ at each subject’s individual threshold, as shown in Fig. 6. Data are shown for each subject separately (S1-S7) and as a mean of all subjects. Q, Mon., and Bin. indicates data for SAM presented in quiet, in monaurally presented notched noise, and in binaurally presented notched noise, respectively, and are shown in blue, purple, and red markers, respectively. The black dashed line indicates chance level, at 0.5. Error bars represent 95% confidence intervals and boxplots indicate median, 25^th^ and 75^th^ percentiles, and outliers, across subjects. *P*-values indicate the outcome of a χ-square test comparing the monaural to the binaural proportion correct. The highest *p*-value of the two tests (two monaural conditions compared to the binaural condition) is shown in the graph. ITD_ENV_ envelope interaural time difference, ΔIPD_ENV_ difference in envelope interaural phase difference between two binaural stimuli, SAM sinusoidally amplitude modulated.

For the second part of the psychophysical study, SAM tones were presented binaurally in quiet (*Q*), in monaurally presented notched noise (*Mon*.), and in binaurally presented notched noise (*Bin*.). SAM tones were presented for each subject at their individual ∆ITD_ENV_ threshold, as derived from the first study (Fig. 6). As expected, the SAM tone in quiet was nearly always correctly lateralized at the individually determined ∆ITD_ENV_, as indicated with a proportion correct ≥0.95 for each subject (*Q* in Fig. 7). Furthermore, most of the subjects replicated a proportion correct that was close to the theoretical 0.84 in the condition with a binaurally presented notched-noise masker (*Bin*. in Fig. 7). Subject S2 had a higher proportion correct compared to the first part of the study, while subjects S4 and S6 dropped in sensitivity. For all subjects, the proportion correct was significantly worse (*p* ≤ 0.01 following a χ-square test) for the conditions with only a monaural notched-noise masker compared to their performance in binaural notched noise (*Mon*. in Fig. 7). Furthermore, three out of seven subjects (S4, S6, and S7) had a proportion correct for the monaural notched noise masker conditions which was not significantly above chance.

These results show a lower discrimination performance in the asymmetric case compared to the quiet or binaural notched-noise conditions, supporting the hypothesis that notched noise shifts the spectral operating point for envelope encoding from off-frequency to on-frequency channel, i.e., to the notch center. As with most psychophysical studies, these results should not be seen as conclusive proof of the hypothesis. Alternative explanations that the applied asymmetry can cause a degradation in ITD sensitivity are possible. For example, as argued, the notched noise can cause a change in amplification, effectively creating an “interaural” level difference that can impair ITD sensitivity. It was not possible to add control conditions that would fully eliminate alternative explanations, because the required low-level or narrow-notch conditions would further restrict the fraction of participants able to obtain stable thresholds in all control conditions.

## Discussion

In the current study, we showed that the envelope of low-level amplitude-modulated tones was encoded in the on-frequency channel, i.e., by the fibers tuned at or close to the carrier frequency. Increasing the level of the SAM tones to levels typical for everyday listening situations (60 to 70 dB SPL) decreased temporal coding in the on-frequency channel drastically, confirming previous physiological recordings in cat, guinea pig, and gerbil^11–15^. In addition, the responses of the modeled on-frequency auditory nerve fiber agreed well with those of the in vivo recorded auditory nerve fibers (Fig. 1), further validating the responses to the temporal envelope in the computational model by Bruce, et al.^19^. Decreased phase locking to the envelope with increasing sound level is explained by rate saturation throughout the cycle of the envelope, resulting from saturation of the inner hair cell receptor potential^16,17^. This phenomenon was clearly observed in both the modeled and the in vivo recorded auditory nerve fibers, as illustrated by the phase histograms shown in Fig. 1d-f. At the higher stimulus level, phase locking to the envelope increased in the off-frequency channels, a signature of off-frequency listening or ‘spread of excitation’ along the cochlear tonotopic array. This is consistent with example data shown for cat auditory-nerve fibers^11,12^ and is supported by similar results from auditory models^21,22^.

Recent computational models have shown that such off-frequency responses are crucial for explaining auditory evoked potentials as well as speech encoding at typical everyday sound levels^17,34^. Specifically, the fluctuation profile model states that a formant of voiced speech is encoded in the auditory nerve by an unmodulated response in the on-frequency channel, i.e., at the formant frequency, and a strongly fluctuating response in the off-frequency channels. These off-frequency channels are phase locking to the fundamental frequency or its lower harmonics. This pattern of activity, and the contrast in fluctuation between the on- and off-frequency responses, is strongest at levels at which we normally communicate, thereby likely aiding in speech comprehension^17^. Experimental recordings of the auditory nerve during the presentation of vowels in quiet confirm that temporal responses to the fundamental frequency and its low harmonics are strongest in auditory nerve fibers outside of the spectral profile of the vowel^7,35^. Together, these results suggest that off-frequency representation of the amplitude modulations are not just an epiphenomenon but serve a function putatively important in auditory perception.

Our auditory nerve modeling results predicted that adding notched noise to the SAM tone should result in an increase of on-frequency phase locking at moderate levels (Fig. 3). This result was qualitatively supported by our auditory nerve recordings. At relative spectral noise levels of −55 dB.Hz^−1^ and −45 dB.Hz^−1^, the model was also quantitatively in line with the data for both notch widths. For a relative spectral noise level of −35 dB.Hz^−1^, the model overestimated the VS improvement (Fig. 4b). This may be explained by a species-specific difference: Tuning at moderate levels (defined by Q_40 dB_) in low-SR fibers, is modeled based on estimated human auditory filters which are sharper than in gerbils^29,36^. In fibers with broader tuning, the notched noise could spill over in the receptive field at 60 dB SPL thereby masking the on-frequency channel as well. Furthermore, an accurate estimate of CF seems crucial for this paradigm. During recording, the CF cannot always be precisely estimated. Thus, the CF of the fiber may not exactly center in the notched noise and the notched noise could partly mask also the response of the recorded fiber, in particular at the highest relative spectral noise level tested (*g* = −35 dB.Hz^−1^). Whereas this effect was attempted to be minimized, we cannot rule out that it may have affected the outcomes.

We hypothesize that the enhanced on-frequency phase locking in the presence of notched noise is similar to the cochlear mechanisms underlying two-tone rate suppression. Indeed, it has been shown that rate suppression can be induced not only by other tones but also by noise, showing similar characteristics as two-tone suppression^18^. As with two-tone suppression, noise suppression also has a strong frequency selective component, meaning that only noise with certain frequencies that are close, but not too close, to the tone at CF can suppress the fiber’s rate response. Correspondingly, in our experiments, the effect of notched noise on envelope coding differed with notch width (Fig. 4). This frequency selective rate suppression is known to originate from mechanical phenomena at the basilar membrane and the role of outer hair cell active gain for rate suppression is evident^37–39^. Similarly, periodic rate suppression by low-frequency biasing also has these similar characteristics, as seen in the motion of the basilar membrane and in the inner hair receptor potentials^37,40,41^. Indeed, our modeled and recorded auditory nerve fibers exhibited both a rate reduction at a range of levels as well as a decreased sensitivity when SAM tones were presented in notched noise of a fixed level (Fig. 5). To sum up, reduced cochlear gain is apparent in our experimental data and could also be underlying the enhanced on-frequency phase locking with notched noise. Such cochlear gain can be further modulated by the medial olivocochlear efferent system whose neurons provide inhibitory synaptic input to the outer hair cells^42^. Our stimulus paradigm involved fairly long stimuli (2.4–3 s, see Table 1) and could, in principle, activate efferent feedback loops that might thus account for part of the observed improvement in on-frequency representation of the stimulus envelope. Whether this is likely under our experimental conditions remains unknown. Efferent activity is believed to be reduced under general anesthesia (reviewed by Guinan, 2011) and possibly abnormal in other ways, considering the potential top-down inputs to the efferent neurons from extensive brain networks^43^. However, efferent modulation is not necessary to explain the principal effect we observed. The computational model used here does not take the effect of the efferent feedback system into account, yet still predicted the observed effect of notched noise on auditory nerve responses. In psychophysical studies with healthy, awake subjects, the olivocochlear efferent system and, more broadly, all brain networks, are, of course, assumed to be fully functional. Thus, our psychophysical data more likely include the modulating effect of top-down efferent networks.

**Table 1:** Stimulation parameters that differed between the three experimental approaches

	Modeling	Auditory nerve recordings	Psychoacoustics
Carrier frequency *f*_*c*_	4 kHz	CF	4 kHz
SAM duration	3 s	2.4 s	0.3 s
Notch width *w* in % of *f*_*c*_	20 and 30%	20 and 30%	20%
SAM level	0 to 90 dB SPL	10 to 81 dB SPL	65 dB SPL
Relative spectral noise level *g*	−65 to 25 dB.Hz^−1^	−55 to 15 dB.Hz^−1^	−35 dB.Hz^−1^
Low- and high frequency cut-offs of the notched noise	1–8500 Hz	300–16,300 Hz	1–8500 Hz

*CF* characteristic frequency, *f*_*c*_, carrier frequency of the SAM tone, *SAM* sinusoidally amplitude-modulated tone, *SPL* sound pressure level.

Notched noise is a workhorse for hearing research, audiologic diagnostics^44,45^, and tinnitus therapy^46^. The role of notched noise in hearing research can be further subdivided into studies that use it to study the auditory filter shape and bandwidth^47,48^ and studies that use it - as we did here - to prevent off-frequency listening^26,27,49,50^. A general problem in both subdivisions, is that the notched noise influences the state of the whole system, including, for instance, the gain and the even the filter shape. The stimulus changes the properties of the system it aims to investigate. Consequently, many of the results drawn from such studies are confounded by the non-linear processing that violates the studies’ basic, crucial assumption. We highlighted this with the example of ITD sensitivity, where the simplified fixed-filter assumption has led to an apparent contradiction between a neurophysiological finding (on-frequency temporal envelope encoding declines with increasing sound level) and a psychoacoustic finding (on-frequency envelope ITD sensitivity increases with level). We showed that the notched noise can shift the level of best envelope phase locking from 20 to 30 dB SPL to as much as 60 dB SPL, an effective change of operating point of about 35 dB. This shift provides a unifying explanation for the otherwise contradictory findings, while emphasizing that each statement is only true in the respective absence or presence of notched noise and possibly only for this specific notched noise.

Generalizing even further, the spectro-temporal composition of any sound creates its unique state for the outer hair cells and all other non-linear or time-dependent processing stages. In principle, this is long known, it has been described in detail for two-tone suppression paradigms^38^, and even incorporated in some auditory processing models^19,20,51^. The present study highlights its practical relevance and contributes experimental data that re-connect psychoacoustic data interpretation with neurophysiological data and supports a state-of-the-art auditory model^19^. As most models of speech-in-noise^52–54^ and model-based audiologic diagnostics^55,56^ are also based on essentially linear time-invariant filter assumptions, care has to be taken when interpreting or even generalizing their simulation results or conclusions.

Furthermore, our findings support the importance of and need for physiologically inspired hearing aid processing, which can restore frequency selectivity and dynamic range of individuals with hearing impairment^57^. Translating the concept of dynamic cochlear amplification to pathological conditions, we suggest that outer hair cell damage likely affects the enhanced envelope coding in noise. This may be a possible mechanism explaining the effect of outer hair cell damage on speech-in-noise perception^2^. In the case of an asymmetric hearing loss, pathologically changed amplification would result in a frequency mismatch of information in both ears, making localization more challenging^23^.

## Methods

### Acoustic stimuli

The stimuli used in all three experiments (computational modeling, single-unit auditory nerve recordings, and human psychoacoustics) were sinusoidally amplitude-modulated (SAM) tones with a fixed modulation frequency *f*_*m*_ = 128 Hz at full modulation depth of 100%. SAM tones were presented in quiet and in spectrally-flanking notched noise. When notched noise was presented to both ears it was interaurally uncorrelated. The notch was linearly centered at *f*_*c*_, with notch width *w* given in % of *f*_*c*_, e.g., a notch width of *w* = 20% ranged from 0.9 *f*_*c*_ to 1.1 *f*_*c*_. On- and off-ramps of the SAM tones were 20 ms. The remaining parametrization of the acoustic stimuli differed within and between the different experiments, as listed in Table 1 and explained in more detail in the respective Method subsections.

### Computational modeling

#### Computational model of the auditory nerve

We used the auditory periphery model proposed by Bruce, et al.^19^, which receives a pressure signal as input and outputs a sequence of auditory nerve spikes. The effect of the middle ear is modeled as a band-pass filter. After this, the signal is processed through three parallel feed-forward paths known as component 1, component 2, and control path, as described in Zilany and Bruce^58^. These pathways capture the response properties of the basilar membrane (BM), inner hair cells, and the outer hair cells. The filtered signal is then converted into receptor potentials of inner hair cells, each with a specific CF. Additionally, the model includes a physiologically accurate representation of the synapses between inner hair cells and auditory nerve fibers. The output of the model is represented by a spike generator that produces a sequence of auditory nerve spikes. For a more comprehensive explanation of the model, please refer to the original paper^19^.

#### Parametrization of the model

In total, we simulated 26 different inner hair cells and auditory nerve fibers with CFs in the range of 2 to 7.5 kHz distributed equidistantly along the tonotopic axis, according to ref. ^59^. For each inner hair cell, one auditory nerve fiber was simulated. We used a basilar membrane tuning optimized for humans^36^. The model was probed with an assumed spontaneous rate of 4 spikes.s^-1^. The relative refractory time was set to *t*_*rel*_ = 513 µs and the absolute refractory time to *t*_*abs*_ = 450 µs; for value range, see Miller, et al.^60^ and Bruce, et al.^19^. The model was implemented in MATLAB (MathWorks, Natick, MA; version R2020).

#### Stimuli and procedure for modeling

Stimuli had a sampling rate of 100 kHz. The SAM tone with a fixed *f*_*c*_ of 4 kHz was presented simultaneously with the notched noise, both with a duration of 3 s including 20-ms cos^2^ rise-decay ramps. The model received 10 repetitions of each stimulus. The aims of the computational modeling part of this work were to determine:

1. The on-frequency rate and VS, as a function of level in quiet (Fig. 1a and Fig. 1d) and in notched noise with *w* = 20% (800 Hz for the *f*_*c*_ = 4 kHz) (Fig. 5a). In this experiment, the SAM tone was presented at an SPL of 0 to 90 dB in steps of 5 dB, while the notched-noise spectral level of 25 dB SPL.Hz^−1^ remained unchanged.
2. The rate and VS across modeled fibers with a different CF, for SAM tones in quiet and in notched noise with *w* = 20%, and a spectral level of 30 dB SPL.Hz^−1^. In this experiment, the SAM tone was presented at an SPL of 20 and 65 dB (Figs. 2 and 3). The *f*_*c*_ remained unchanged, fixed at 4 kHz.

#### Data analysis

The degree of phase locking can be quantified by the vector strength (VS)^61^. Each individual spike is represented as a unit vector with angle *α*_*k*_ corresponding to the spike time within the modulation cycle. The VS is defined as

$${VS}=\left|\frac{1}{K}{\sum }_{k=1}^{K}{e}^{i{\alpha }_{k}}\right|$$

with *K* being the total number of spikes measured for a given condition and *k* indicating the *k*th spike. If all spikes occur at a single phase of the modulation cycle, *VS* will be equal to 1. Conversely, if spike timing is random within the modulation cycle, *VS* approaches 0. The *VS* was tested for significance by calculating the *p*-value as follows

$$p={e}^{-K.{{VS}}^{2}}$$

When *K* > 50 and *p* < 0.001, *VS* was considered significant^62^. Non-significant *VS* values are presented but without a marker in Figs. 1 to 4. The spike rate and *VS* was calculated for the 10 repetitions for each CF and averaged afterwards.

### Auditory nerve single-unit recordings in gerbils

#### Experimental Procedures

Single-unit recordings were collected from the auditory nerve of nine young-adult (3 to 6 months), normal-hearing Mongolian gerbils, *Meriones unguiculatus* (four females). Gerbils were born at the University of Oldenburg animal house and were group housed in a cage with environmental enrichment in a climate controlled, quiet environment. We have complied with all relevant ethical regulations for animal use. Experimental procedures were reviewed and approved by the ethics authorities of Lower Saxony (LAVES), Germany, by the permit numbers AZ 33.19-42502-04-15/1990 and AZ 33.19-42502-04-21/3695. There was no protocol registered before the start of this study.

Surgical procedures and single unit recordings are described in detail by ref. ^63^. Briefly, animals were anesthetized with 135 mg.kg^-1^ ketamine (Ketamin 10%, Ketamidor, WDT) and 6 mg.kg^-1^ xylazine (Xylazin 2%, Serumwerk), and received additional oxygen of 1.5 L.min^-1^ in front of the snout throughout the experiment. In a subset of animals (6 of 9), meloxicam, a non-steroidal antiphlogistic agent (1 mg/kg; Metacam 2 mg/ml, Boehringer Ingelheim), was injected at the beginning of the experiment. The heartbeat, breathing, and muscle potentials of the animal were constantly monitored on an electrocardiogram and body temperature was kept constant at 38 °C. Stimuli were presented in a closed field by a small speaker (IE 800, Sennheiser) that was sealed in an ear bar together with a miniature microphone (ER7-C, Etymotic Research) to calibrate the stimuli. The ear bar was sealed to the bony ear canal and a small opening in the dorsal-lateral bulla prevented negative pressure buildup in the middle ear cavity. The auditory-nerve bundle was approached dorsally by partial aspiration of the cerebellum and the placement of small saline-drenched paper balls between the brainstem and the temporal bone. Recordings were carried out in a custom-built sound-attenuating chamber. The experiment was terminated when breathing or heart rate of the animal became irregular or when hearing thresholds were elevated.

#### Data collection

Single-unit recordings were made with the use of glass micropipette electrodes (BF120F-10, Science Products; pulled on a P-2000 puller, Sutter Instruments, Co.), that were filled with 3 M KCl solution and had a typical impedance between 20 and 40 MΩ. Broadband noise bursts (50 to 70 dB SPL) were presented while the electrode was slowly advanced through the bundle (1 to 5 µm steps; inchworm motor controller, 6000 ULN Burleigh) to isolate single fibers. Recorded signals were amplified (10x, WPI 767), filtered for line-frequency noise (50/60 Hz; Hum Bug, Quest Scientific), made audible through a speaker (MS2, TDT), visualized on an oscilloscope (SDS 1102CNL, SIGLENT Technologies), and digitized (RX6, TDT; 48,828 Hz sampling rate) before being displayed and stored using custom-written MATLAB software.

After a single unit was isolated, tone bursts (50-ms duration, 5-ms cosine ramps, 5 repetitions) at a range of frequencies and at a fixed level of 10 dB above the estimated threshold were presented to determine the unit’s CF. Next, tone bursts (50-ms duration, 5-ms cosine ramps, 10 repetitions) with a range of stimulus levels at CF were presented to derive the unit’s firing rate threshold. Subsequently, SAM tones in quiet and in notched noise, as described above (Stimuli and Table 1), were presented (*f*_*c*_ = CF, 2.4-s duration, 20-ms cos^2^ ramps, 10 repetitions, 48,828 Hz sampling rate). All stimuli were calibrated using custom-made MATLAB software for each animal individually.

#### Data analysis

Raw voltage traces were bandpass filtered (300 to 3000 Hz) and spikes were identified using a simple voltage detection threshold that was established on a trial-by-trial basis. CF was determined from the frequency-response curve as its highest peak and threshold was determined from the rate-level function as the lowest stimulus level yielding a significant increase in firing rate. SR was estimated from a 24-s recording in quiet. When this was not available (*n* = 7 of 24 units), silent trials recorded during the collection of the rate-level function were used to estimate SR (800 ms total duration). All fibers included in this study were checked for typical auditory nerve fiber characteristics (e.g., absence of pre-potential to the spike waveform, typical rate-level function and peri-stimulus time histogram shape, and click latency conform with auditory nerve). For responses to the 2.4 s SAM tones in quiet and in notched noise, the analysis window was defined from 20 ms after stimulus onset, to exclude the initial onset response, until stimulus offset. The VS to *f*_*m*_ of the SAM tones was calculated over the spikes that fell into the analysis window and tested for significance as described above (Computational modeling – Data analysis). Only significant VS values (*p* < 0.001 and *K* > 50) were included in the analyses. The same analysis window was also applied when calculating average firing rate.

### Psychoacoustic experiments with humans

#### Subjects

A total of 12 normal-hearing subjects participated in the first psychophysical experiment, determining individual ΔIPD_ENV_. Five out of these 12 subjects were excluded as they could not perform the task or had no converging localization thresholds. The remaining seven subjects (4 female, aged between 21 and 29 years) had audiometric thresholds equal or less than 25 dB HL at octave-spaced frequencies from 125 to 8000 Hz (see Supplementary Fig. 3). The total duration of the measurements was 2 to 3 h per subject, performed in two sessions. Within a 45-to-90-min session, subjects could take as many breaks as they wanted. All ethical regulations relevant to human research participants were followed. The study was approved by the Ethics committee of the University of Oldenburg and participants provided written informed consent.

#### Procedure

The procedure was a two-interval, two-alternative forced-choice task (2I-2AFC). The task of the subject was to indicate whether the SAM tone in the second interval was perceived to the left or the right of that presented in the first interval. The stimuli had synchronous onset and offset gating in both ears, so that the SAM tones differed only in their ongoing interaural phase difference of the envelope (IPD_ENV_). SAM tones had a carrier frequency *f*_*c*_ of 4 kHz and were presented to the subject via Sennheiser HD-650 headphones at a SPL of 65 dB. Levels where calibrated using a sound-level meter and an Artificial Ear Type 4153 (Brüel & Kjær). Digital-analog conversion was carried out by ADI-s DAC FS (RME) with 32 bit and a 48-kHz sampling rate. Stimuli were generated digitally using the AFC-software package^64^. The subjects were seated in a double-walled, sound-attenuating booth and responded by pressing a key on a standard computer keyboard. Visual feedback was provided after each trial. The 300-ms SAM tone duration included 20-ms cos^2^ rise-decay ramps. A 50-ms silent interval separated the two intervals. A next pair of intervals was presented 500 ms after the subject responded. The IPD_ENV_ of the stimuli presented in the two intervals were symmetrical around zero^65–67^, so that in one of the two intervals, the right ear was leading, in the other the left ear led by the same IPD_ENV_. Subjects could thus make their decision based on a ΔIPD_ENV_ ( = 2 IPD_ENV_ used in the individual intervals) difference between the two intervals.

The experiment consisted of two phases. In the first phase, the threshold IPD-value was estimated for the baseline condition (with spectrally-flanking notched noise presented to both ears with no interaural correlation). An adaptive ‘four-down, one-up’ staircase procedure controlled the ΔIPD_ENV_, meaning that the ΔIPD_ENV_ was decreased after four correct responses in a row and increased after each incorrect response, adapting towards a proportion correct of 0.84^32^. This target rate of 0.84 provides a large result space for a deterioration before a floor is hit at chance level 0.5. Each adaptive track started at a ΔIPD_ENV_ of 0.4π radians. The maximum allowed ΔIPD_ENV_ value was 0.8π, because a decline of sensitivity is expected for higher values^33^. With this upper limit for the adaptive tracking variable, a slightly lower upper bound of approximately 0.7π radians, is expected for the mean values. The initial step size was a factor of 2, which was reduced to 1.414 (2^1/2^) and 1.189 (2^1/4^) after the first and second ‘down-up-reversal’. An adaptive track was terminated after 10 reversals at the minimum step size. This sequence was repeated three times for each subject.

In the second part, the proportion correct for a fixed ΔIPD_ENV_ was measured in four different conditions: (1) in quiet, (2, 3) in spectrally-flanking notched noise at only the left or right headphone channel, and (4) in a control condition with the same interaurally uncorrelated notched noise as in the first part.

For the second, third, and fourth conditions, the spectrally-flanking noise was presented continuously, to minimize binaural interference^68^, in line with Bernstein and Trahiotis^27^. The ΔIPD_ENV_ was the geometric mean of the thresholds from the first part. The spectrally-flanking notched noise had a spectral level of −35 dB relative to the SAM tone. The notch was centered at 4 kHz and had a width of 800 Hz between 3600 and 4400 Hz (*w* = 20%). To avoid the use of low-frequency distortion products arising from nonlinear peripheral auditory processing^23^, a continuous diotic noise, low passed at 1.3 kHz with a relative spectral level of *g* = −35 dB.Hz^−1^ was added in all conditions^27^.

### Statistics and reproducibility

Data were tested for normality using the Anderson-Darling test. When the test was significant (*p* < 0.05), the data were statistically examined using non-parametric tests, otherwise parametric tests were applied. When applicable, statistical tests were two-sided. Bonferroni-Holm corrections were used to correct for multiple comparisons. Statistical analyses were carried out in MATLAB (MathWorks; version R2023b) using the Statistics and Machine Learning Toolbox™ (version 23.2). Statistical tests were applied to data from *n* = 24 recorded single-unit auditory nerve fibers and *n* = 7 human participants.

### Reporting summary

Further information on research design is available in the Nature Portfolio Reporting Summary linked to this article.

## Supplementary information

Supplemental Material

Reporting Summary

## Data Availability

The model outcomes can be reproduced by using the parametrization and stimulus characteristics, as detailed in the Methods section, and applying these to the model listed in the Code Availability section. Single-unit auditory nerve fiber responses were added to our online database for gerbil auditory nerve fiber recordings^69^: 10.5061/dryad.qv9s4mwn4. Anonymized data from the psychoacoustic experiments can be downloaded from Zenodo^70^, using the following link: 10.5281/zenodo.15005128. Source data underlying the graphs with modeled and recorded auditory nerve fiber activity (Figs. 1–5) is shared on Figshare and can be accessed using the following link: 10.6084/m9.figshare.29069324.

## Code availability

The code for the computational model by Bruce, et al.^19^ was downloaded from https://www.ece.mcmaster.ca/~ibruce/zbcANmodel/zbcANmodel.htm. Parametrization and stimulus characteristics used to run the code can be found in the Methods section. Code that was used to analyze the auditory nerve fiber responses are published on Zenodo^70^, through the following 10.5281/zenodo.10370063.
