# Supplementary material for: Notched noise reveals differential improvement in the neural representation of the sound envelope
Source: Commun Biol. 2025 Aug 7;8:1171. doi: 10.1038/s42003-025-08536-4 (PMC12328572; doi:10.1038/s42003-025-08536-4)
Supplement: Supplementary file 1 — Supplemental Material [file 42003_2025_8536_MOESM1_ESM.pdf]

Supplementary Figure 1

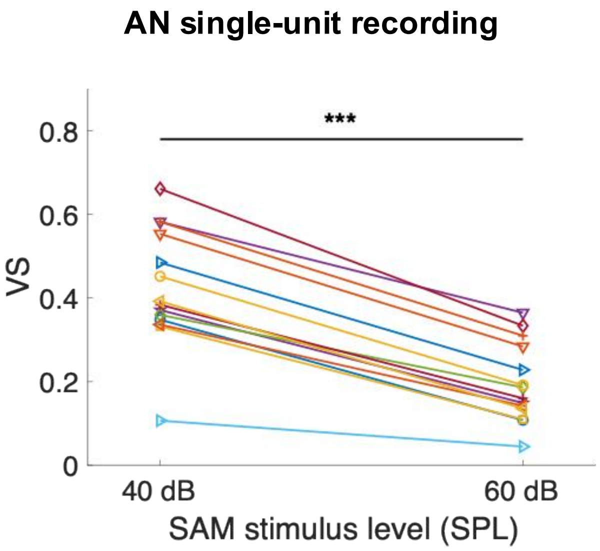

Supplemental Figure 1. On-frequency phase locking to the stimulus envelope decreases with level. Phase locking, expressed as VS, plotted as a function of the stimulus level of the SAM tone, derived from n = 14 single-unit recordings in AN fibers of the gerbil. The carrier frequency of the SAM tone was similar to each fiber's CF. Each marker and color combination represents data derived from one recorded fiber, corresponding to the ones used in Fig. 4. *** indicates $p<0.001$, following a one-sample Student's T-test.
AN, auditory nerve; CF, characteristic frequency; SAM, sinusoidally amplitude modulated tone; SPL, sound pressure level; VS, vector strength.

Supplementary Figure 2

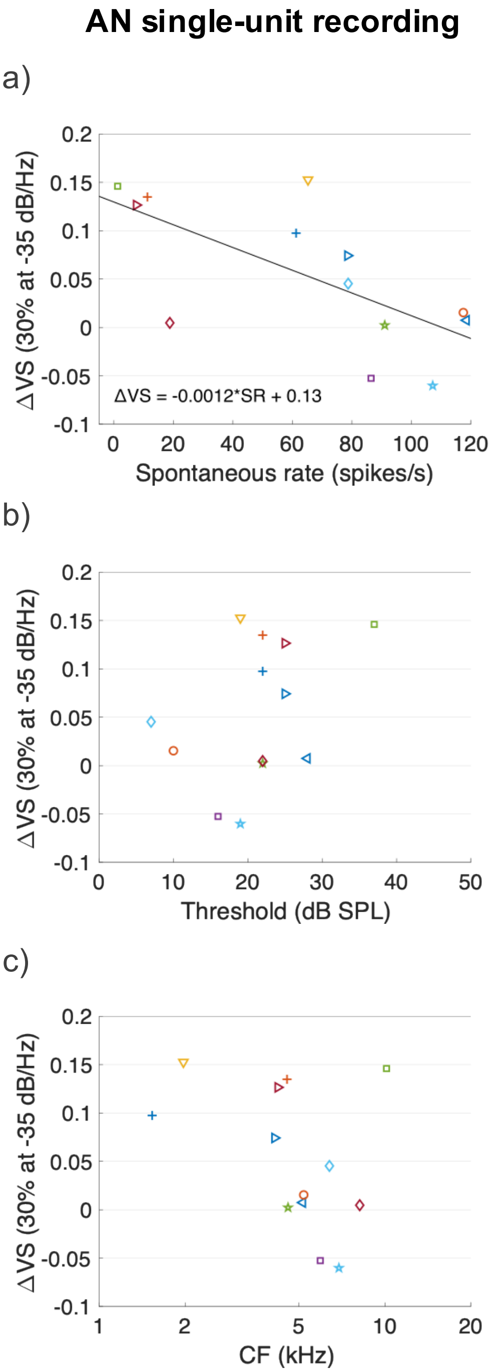

Supplemental Figure 2. Relations between notched noise-induced changes in VS and fiber characteristics. a) Change in VS (ΔVS) by notched noise (notch width *w* = 30%, relative spectral noise level *g* = -35 dB.Hz^{-1}) plotted as a function of SR. The black line represents the linear regression between SR and ΔVS, which is indicated in the graph. Each marker and color combination represents data derived from one recorded fiber and corresponds to the same marker and color combination used in Fig. 4. b) ΔVS plotted as a function of the fiber's pure-tone threshold. The correlation analysis was not significant (Pearson's correlation coefficient $\rho = 0.39$, $p = 0.183$). c) ΔVS plotted as a function of CF. The correlation analysis, carried out on log(CF), was not significant ($\rho = -0.40$, $p = 0.181$).

AN, auditory nerve; CF, characteristic frequency; SPL, sound pressure level; SR, spontaneous rate; VS, vector strength.

Supplementary Figure 3

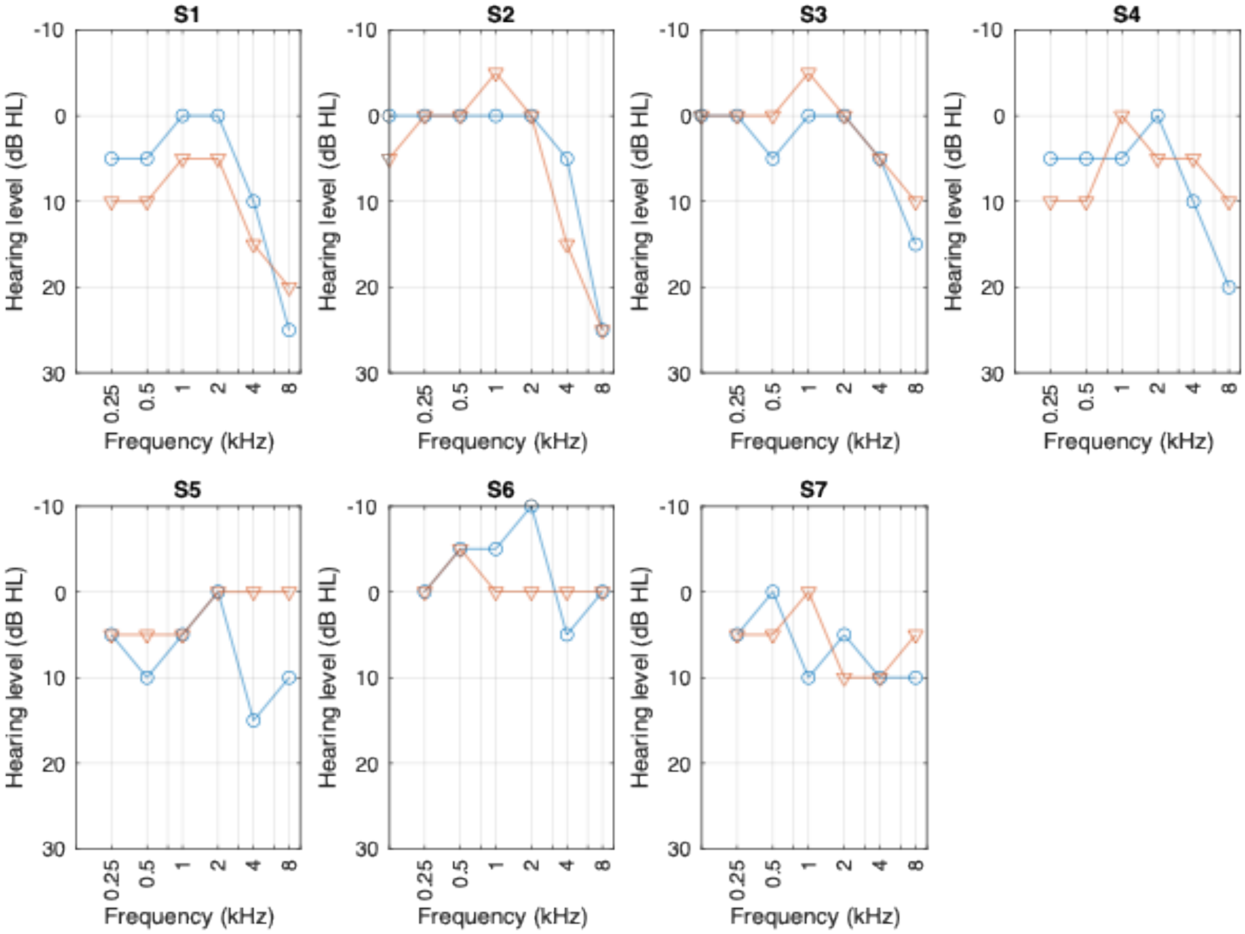

Supplemental Figure 3. Audiograms of the individual subjects. Hearing thresholds, in dB HL, for each of the seven included subjects. Thresholds of the left ear are in blue circles, thresholds of the right ear are in red downward triangles. Octave-spaced frequencies between 125 and 8000 Hz were tested. Subject IDs (S1 – S7) are similar to subject IDs in Fig. 6 and Fig. 7.
