# Supplementary material for: Notched noise reveals differential improvement in the neural representation of the sound envelope
Source: Commun Biol. 2025 Aug 7;8:1171. doi: 10.1038/s42003-025-08536-4 (PMC12328572; doi:10.1038/s42003-025-08536-4)
Supplement: Supplementary file 2 — Reporting Summary [file 42003_2025_8536_MOESM2_ESM.pdf]

nature portfolio

Corresponding author(s): Mathias Dietz

Last updated by author(s): Jul 10, 2025

Reporting Summary

Nature Portfolio wishes to improve the reproducibility of the work that we publish. This form provides structure for consistency and transparency in reporting. For further information on Nature Portfolio policies, see our Editorial Policies and the Editorial Policy Checklist.

Statistics

For all statistical analyses, confirm that the following items are present in the figure legend, table legend, main text, or Methods section.

n/a	Confirmed	
☐	☒	The exact sample size (*n*) for each experimental group/condition, given as a discrete number and unit of measurement
☐	☒	A statement on whether measurements were taken from distinct samples or whether the same sample was measured repeatedly
☐	☒	The statistical test(s) used AND whether they are one- or two-sided<br>*Only common tests should be described solely by name; describe more complex techniques in the Methods section.*
☒	☐	A description of all covariates tested
☐	☒	A description of any assumptions or corrections, such as tests of normality and adjustment for multiple comparisons
☐	☒	A full description of the statistical parameters including central tendency (e.g. means) or other basic estimates (e.g. regression coefficient) AND variation (e.g. standard deviation) or associated estimates of uncertainty (e.g. confidence intervals)
☐	☒	For null hypothesis testing, the test statistic (e.g. *F*, *t*, *r*) with confidence intervals, effect sizes, degrees of freedom and *P* value noted<br>*Give P values as exact values whenever suitable.*
☒	☐	For Bayesian analysis, information on the choice of priors and Markov chain Monte Carlo settings
☒	☐	For hierarchical and complex designs, identification of the appropriate level for tests and full reporting of outcomes
☒	☐	Estimates of effect sizes (e.g. Cohen's *d*, Pearson's *r*), indicating how they were calculated

Our web collection on statistics for biologists contains articles on many of the points above.

Software and code

Policy information about availability of computer code

Data collection	Modeled data was collected using the publicly available code for the computational model (https://www.ece.mcmaster.ca/~ibruce/zbcANmodel/zbcANmodel.htm). Single-unit recordings were collected using a custom-written software package running in MATLAB and using RPvdsEx circuits to drive the TDT hardware. Human psychophysical data was collected using a custom-written software in Matlab.
Data analysis	Code that was used to analyze the auditory nerve fiber responses are published on Zenodo, through the following DOI: https://doi.org/10.5281/zenodo.10370063.<br>Code that was used to analyze and plot the psychophysical data is available on https://doi.org/10.5281/zenodo.15005128

For manuscripts utilizing custom algorithms or software that are central to the research but not yet described in published literature, software must be made available to editors and reviewers. We strongly encourage code deposition in a community repository (e.g. GitHub). See the Nature Portfolio guidelines for submitting code & software for further information.

Data

Policy information about availability of data

All manuscripts must include a data availability statement. This statement should provide the following information, where applicable:

- Accession codes, unique identifiers, or web links for publicly available datasets
- A description of any restrictions on data availability
- For clinical datasets or third party data, please ensure that the statement adheres to our policy

The model outcomes can be reproduced by using the parametrization and stimulus characteristics, as detailed in the Methods section, and applying these to the model listed in the Code Availability section. Single-unit auditory nerve fiber responses are added to our online database for gerbil auditory nerve fiber recordings: https://doi.org/10.5061/dryad.qv9s4mwn4. Anonymized data from the psychoacoustic experiments can be downloaded from Zenodo, using the following link: https://doi.org/10.5281/zenodo.15005128

Research involving human participants, their data, or biological material

Policy information about studies with human participants or human data. See also policy information about sex, gender (identity/presentation), and sexual orientation and race, ethnicity and racism.

Reporting on sex and gender	Sex is reported and balanced. It was not expected to play a role and indeed all 3 male and all 4 female participants show the effect.
Reporting on race, ethnicity, or other socially relevant groupings	Not reported or collected. It was not expected to play a role. We even show consistency across species and reasonably expect the effect to be qualitatively the same across mammalian species.
Population characteristics	Again, as we only report the existence of an effect clearly in 7 of 7 normal-hearing participants, quantitative analysis which would require such characteristics is out of the scope of the study.
Recruitment	We recruited mostly university students through a University online portal and word of mouth.
Ethics oversight	Ethics committee of the University of Oldenburg

Note that full information on the approval of the study protocol must also be provided in the manuscript.

Field-specific reporting

Please select the one below that is the best fit for your research. If you are not sure, read the appropriate sections before making your selection.

☒ Life sciences ☐ Behavioural & social sciences ☐ Ecological, evolutionary & environmental sciences

For a reference copy of the document with all sections, see nature.com/documents/nr-reporting-summary-flat.pdf

Life sciences study design

All studies must disclose on these points even when the disclosure is negative.

Sample size	No sample size calculation was applied, since this paper focuses on a 'proof-of-principle'. It shows the presence of a phenomenon in the phaselocking ability of auditory nerve fibers, rather than the extent of it in multiple situations or treatments.
Data exclusions	5 of 12 human subjects were excluded as they could not perform the task. This was expected and numbers are reported. Recordings from gerbil auditory nerve fibers were excluded when they did not comply with typical auditory nerve fiber characteristics.
Replication	We replicated our model predictions in 24 single-unit auditory nerve fibers recorded from Mongolian gerbils.
Randomization	There were no separate treatment groups, therefore randomization was not relevant for this study. All conditions were tested in all participants and in all single fibers.
Blinding	Blinding was not relevant to this study, as all the analyses were objective and no treatment was provided.

Reporting for specific materials, systems and methods

We require information from authors about some types of materials, experimental systems and methods used in many studies. Here, indicate whether each material, system or method listed is relevant to your study. If you are not sure if a list item applies to your research, read the appropriate section before selecting a response.

Materials & experimental systems

n/a	Involved in the study
☒	☐ Antibodies
☒	☐ Eukaryotic cell lines
☒	☐ Palaeontology and archaeology
☐	☒ Animals and other organisms
☒	☐ Clinical data
☒	☐ Dual use research of concern
☒	☐ Plants

Methods

n/a	Involved in the study
☒	☐ ChIP-seq
☒	☐ Flow cytometry
☒	☐ MRI-based neuroimaging

Animals and other research organisms

Policy information about studies involving animals; ARRIVE guidelines recommended for reporting animal research, and Sex and Gender in Research

Laboratory animals	Mongolian gerbils (Meriones unguiculatus), 3 - 6 months of age.
Wild animals	The study did not involve wild animals.
Reporting on sex	Data derived from four female Mongolian gerbils, sex was not expected to be a factor in the outcomes of this study.
Field-collected samples	The study did not involve samples collected from the field.
Ethics oversight	The ethics authorities of Lower Saxony, Germany (LAVES)

Note that full information on the approval of the study protocol must also be provided in the manuscript.

Plants

Seed stocks	*Report on the source of all seed stocks or other plant material used. If applicable, state the seed stock centre and catalogue number. If plant specimens were collected from the field, describe the collection location, date and sampling procedures.*
Novel plant genotypes	*Describe the methods by which all novel plant genotypes were produced. This includes those generated by transgenic approaches, gene editing, chemical/radiation-based mutagenesis and hybridization. For transgenic lines, describe the transformation method, the number of independent lines analyzed and the generation upon which experiments were performed. For gene-edited lines, describe the editor used, the endogenous sequence targeted for editing, the targeting guide RNA sequence (if applicable) and how the editor was applied.*
Authentication	*Describe any authentication procedures for each seed stock used or novel genotype generated. Describe any experiments used to assess the effect of a mutation and, where applicable, how potential secondary effects (e.g. second site T-DNA insertions, mosiacism, off-target gene editing) were examined.*
